# The pioneer cohort of curriculum reform: Guinea pigs or trail-blazers?

**DOI:** 10.1186/1472-6920-5-26

**Published:** 2005-07-15

**Authors:** Michelle McLean

**Affiliations:** 1 Department of Physiology, Nelson R. Mandela School of Medicine, University of KwaZulu-Natal, Durban, South Africa

## Abstract

With curriculum reform, whether we admit it or not, the first cohort of students will be 'test-driving' the new programme. Not only are they the pioneers of a new curriculum, but as they progress through their studies, they experience each year of the innovation for the first time. As curriculum designers, we learn from their experiences and their feedback to improve the programme content and delivery, invariably for subsequent cohorts. A considerable onus therefore rests with this pioneer group, and their contribution to curriculum design, evaluation and programme revision should be valued.

## Text

With curriculum reform, whether we admit it or not, the first cohort of students will be 'test-driving' the new programme. Not only are they the pioneers of a new curriculum, but as they progress through their studies, they experience each year of the innovation for the first time. As curriculum designers, we learn from their experiences and their feedback to improve the programme content and delivery, invariably for subsequent cohorts. A considerable onus therefore rests with this pioneer group, and their contribution to curriculum design, evaluation and programme revision should be valued [1,2].

From a personal perspective, based on experiences with our pioneers in an institution with a long traditional history, much advice can be offered to faculties considering implementing a curriculum such as problem-based learning (PBL). Perhaps of most importance is that pioneer students should not feel like experimental subjects or 'guinea pigs', but rather as torch-bearers and ambassadors of the new programme whose constructive input is valued. They should, however, not be overloaded with evaluation, and they must benefit from *their *input into programme revision.

Criticism, rumours and misconceptions are detrimental – address them in the early stages of curriculum reform [3]. To ensure buy-in, involve staff and students in the planning and development of the new programme from the outset, and update them regularly on the progress. Advertising well defined outcomes and objectives by which competency is to be assessed will allay fears regarding quality issues. Also critical is a dean or senior faculty leader who is perceived to be a major player driving the reform [3,4].

Curriculum developers and organisers need clear insight (i.e. long-term planning) into the entire PBL curriculum, particularly if it is of shorter duration than the traditional programme. If registration is not suspended for one year (highly recommended), bear in mind that there will be two cohorts of final year students. Students in both curricula will need reassurance of equitable support in this year.

The year in which the pioneer students are studying must be well organised, with minimal disruption. Major modifications only serve to exacerbate uncertainty and provide sceptics with ammunition for criticism. Each year of the programme must be planned in advance as students *will* ask for details of what they can expect the following year.

As student learning is driven by the assessment and as their progress hinges on their success in the assessment, this aspect of the curriculum must be assigned priority, rather than leaving it to one of the last issues to be addressed in the reform [5]. Explain the format and rationale for the assessment, as it will (or should) be different from what they are accustomed to.

Providing pioneers with sufficient support (e.g. meetings with curriculum developers; assistance with self-directed learning skill; counselling) is of critical importance, as these students have no senior PBL colleagues to consult. Ideally, faculty mentors would be of great benefit to these students, but since the human resource capacity is often limited, interaction between students of the two curricula should be encouraged. Our research suggests that students from different curricula draw on each other's strengths, forming extensive informal student support networks. Faculties should capitalise on these interactions by formalising cross-curriculum peer support groups.

Students deserve the right to contribute to their curriculum. Engaging with pioneers as they progress through their studies provides not only valuable insight into their experiences as trail-blazers but also into those of their traditional curriculum colleagues. Just as pioneers need to be nurtured, traditional curriculum students deserve the same support during the phasing out of their curriculum. Documenting student perceptions during the simultaneous offering of dual curricula is certainly an area for future research as the handful of comparisons between the two curricula have, to date, concentrated on the outcomes (as opposed to the 'process') of programmes.

## Competing interests

The author(s) declare that they have no competing interests.

## Pre-publication history

The pre-publication history for this paper can be accessed here:


